# Highly Efficient Reduction of Cr (VI) with C_4_H_6_O_6_

**DOI:** 10.3390/molecules29225459

**Published:** 2024-11-19

**Authors:** Hao Peng, Zonghui Qin, Guixuan Jin, Jingjing Wang, Jielin Qin, Lihua Ao, Bing Li

**Affiliations:** Chongqing Key Laboratory for New Chemical Materials of Shale Gas, College of Chemistry and Chemical Engineering, Yangtze Normal University, Chongqing 408100, China; 19910013@yznu.edu.cn (Z.Q.); 220923101112@yznu.stu.edu.cn (G.J.); 230923101228@yznu.stu.edu.cn (J.W.); 230210101438@yznu.stu.edu.cn (J.Q.); 230923101101@yznu.stu.edu.cn (L.A.); libing@yznu.edu.cn (B.L.)

**Keywords:** reduction, tartaric acid, Cr (VI)

## Abstract

In this paper, tartaric acid (C_4_H_6_O_6_) was used as a reductant to treat chromium (VI)-containing solution. Several independent experimental parameters, including reaction temperature, concentration of H_2_SO_4_, concentration of C_4_H_6_O_6_ and reaction time, on the reduction process were studied. The results showed that 100% of the Cr (VI) could be reduced by C_4_H_6_O_6_ in a strong acidic environment under a high reaction temperature. All of the experimental parameters showed positive effects on the reduction process and followed the order [H_2_SO_4_] > [C_4_H_6_O_6_] > reaction temperature > reaction time. A higher concentration of tartaric acid and higher reaction temperature could facilitate the reduction process and reduce reaction time.

## 1. Introduction

Chromium (Cr) is an important strategic metal used in many fields due to its excellent physicochemical properties [1,2,3,4,5]. However, excess chromium in the environment is a global threat to global economic development. Cr is one of the most toxic and harmful heavy metals in the environment and been classified into Group I by the IARC [6,7,8]. The existence of Cr in wastewater has brought various diseases which are harmful to life all over the world; thus, the concentration of Cr (VI) in drinking water is strictly limited to below 0.05 mg/L by the WHO [9,10]. In general, Cr has many valences, like Cr (0) in metal used in steels and alloys; Cr (II) in water, seldom seen; Cr (III) in solution and insoluble Cr with a pH range of 6.0 to 9.0; Cr (IV) and Cr (V) merely seen; and Cr (VI) in ions as Cr_2_O_7_^2−^, HCrO_4_^−^ and CrO_4_^2−^. Among them, Cr (VI) and Cr (III) are the most common, and Cr (VI) is more mobile and hazardous than Cr (III) in solutions [11].

In the past decades, many researchers had done their best to promote efficient treatment technologies for chromium removal [12,13,14,15,16,17]. These technologies were mainly classified into three kinds: physicochemical technology, electrochemical technology, and advanced oxidation technology. Physicochemical technologies included membrane filtration, chemical precipitation, ion exchange, and adsorption. Electrocoagulation, electrochemical reduction, electrodialysis, and electrodeionization have belonged to electrochemical technology. Photocatalysis and nanotechnology were advanced oxidation technology, which is a practical approach in treating wastewaters. All of the mentioned technologies above are shown in Table 1 [16,18,19,20,21,22]. A reduction process was involved in the mentioned technologies; it was a common strategy to relieve toxicity and prevent Cr (VI) diffusion and it had attracted much more attention. During the reduction process, Cr (VI) was reduced to Cr (III) and further removed as Cr (OH)_3_ in an alkaline or neutral medium.

Chemical reduction with ferrous metals and precious metals seemed to be an efficient method, while these processes suffered from the leakage of ferrous and precious metals, causing secondary pollution [23,24,25]. Organic matters were capable of reducing Cr (VI) to Cr (III); some low-molecular-weight organic compounds were applied efficiently, like oxalic acid, tartaric acid, and citric acid [26,27,28,29]. Among them, tartaric acid (C_4_H_6_O_6_) was a small molecular organic polycarboxylate with two -COOH and two α-OH groups, which had strong reduced properties and strong complexional ability and were eco-friendly [30,31,32]. Owing to this, it had received widespread attention.

In this paper, the direct reduction of Cr (VI) with C_4_H_6_O_6_ was investigated, and the effects of experimental parameters (reaction temperature, concentration of H_2_SO_4_, concentration of C_4_H_6_O_6_, and reaction time) on the reduction process were studied.

## 2. Results and Discussion

### 2.1. Reaction Mechanism

**Figure 1:**
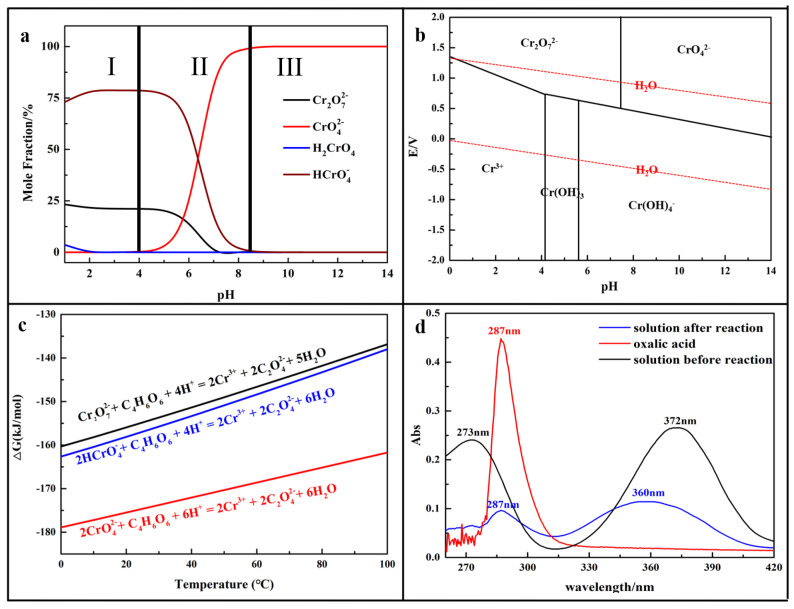
(**a**) Cr (VI) species in Cr (VI)-H_2_O system at 25 °C; (**b**) E-pH diagram for Cr; (**c**) relationship of ΔG-T for main reactions; (**d**) UV-vis spectrum for various solutions.

During the reduction process, the main reactions occurred between Cr (VI) and C_4_H_6_O_6_. The Cr (VI) species that existed in the solution were simulated by the software Visual MINTEQ 3.0 and the results were shown in Figure 1a. In the Cr (VI) solution, the Cr (VI) species were mainly divided into three parts: (I) HCrO_4_^−^, Cr_2_O_7_^2−^ (pH < 4.0); (II) HCrO_4_^−^, Cr_2_O_7_^2−^, CrO_4_^2−^ (4 < pH < 8); and (III) CrO_4_^2−^ (pH > 8). During the reduction process, the solution was kept in acidic medium (Cr_2_O_7_^2−^ was more easily reduced into Cr (III) than CrO_4_^2−^ as the oxidation potential was higher (E^0^ (Cr_2_O_7_^2−^/Cr^3+^) = 1.35 V, E^0^ (CrO_4_^2−^/Cr^3+^) = 0.56 V)) (according to the results shown in Figure 1b); the Cr (VI) species were mainly located in the sections of part I and part II, and the main reactions were expressed as follows:

Cr_2_O_7_^2−^ + C_4_H_6_O_6_ + 4H^+^ = 2Cr^3+^ + 2C_2_O_4_^2−^ + 5H_2_O (1)

2HCrO_4_^−^ + C_4_H_6_O_6_ + 4H^+^ = 2Cr^3+^ + 2C_2_O_4_^2−^ + 6H_2_O (2)

2CrO_4_^2−^ + C_4_H_6_O_6_ + 6H^+^ = 2Cr^3+^ + 2C_2_O_4_^2−^ + 6H_2_O (3)

Meanwhile, the reaction thermodynamics of the above three equations were analyzed by HSC Chemistry 6.0 and the results are shown in Figure 1c. The calculated ΔG were all below zero, indicating that all three reactions were feasible in thermodynamics. The UV-vis spectrum shown in Figure 1d confirms that the reaction happened as in Equations (1)–(3). The peaks at 273 nm and 372 nm correspond to the existence of Cr (VI). The same peak at 287 nm confirms the production of oxalate (C_2_O_4_^2−^) during the reduction process and the peak at 360 nm is attributed to the existence of Cr (III).

### 2.2. Single-Factor Experiments

To better understand the reduction process, the effects of the experimental parameters (concentration of H_2_SO_4_ ([H_2_SO_4_]), concentration of C_4_H_6_O_6_ ([C_4_H_6_O_6_]), reaction time, and reaction temperature) on the reduction process were investigated.

**Figure 2:**
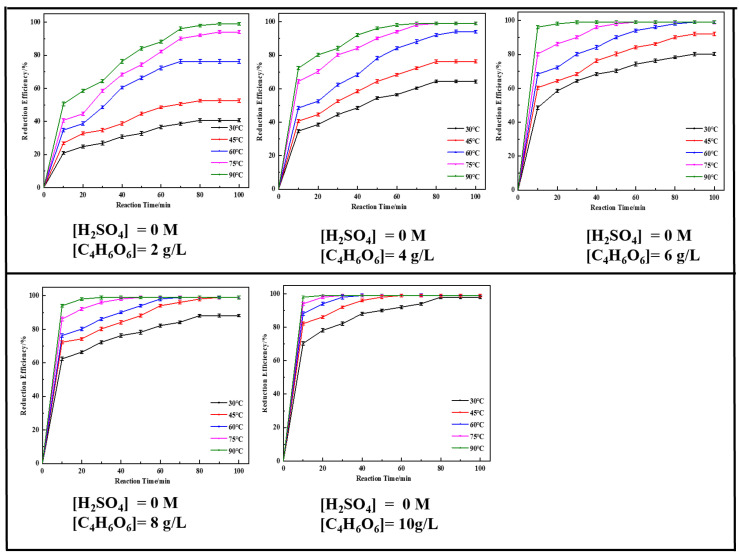
The effect of the experimental parameters on the reduction efficiency of Cr (VI) at [H_2_SO_4_] = 0 M.

The reaction temperature was an important parameter in the chemical process. In this study, the reaction temperature was set as 30 to 90 °C with an interval of 15 °C. The results shown in Figure 2 indicate that the reduction efficiency of Cr (VI) was significantly improved with an increase in reaction temperature, regardless of other reaction conditions. The highest reduction efficiency of 40.63% was obtained at 30 °C for about 80 min, which is similar to the reduction process in soils [33,34]. While it was 50.53% for just 10 min at 90 °C, the highest efficiency of 100% could be achieved. The reduction process was further strengthened by increasing the concentration of C_4_H_6_O_6_. When there was 10 g/L of [C_4_H_6_O_6_], the reduction efficiency was 70.31% at just 10 min, and it increased to 98.02% with a long time reaction at just 30 °C. A total of 100% of Cr (VI) was reduced just in 20 min as the reaction temperature increased to 90 °C. In other words, a higher reaction temperature could reduce the reaction time and achieve a higher reduction efficiency.

As a main reaction reagent, the influence of the concentration of C_4_H_6_O_6_ ([C_4_H_6_O_6_] = 2, 4, 6, 8, and 10 g/L) on the reduction efficiency of chromium was studied. The reduction efficiency increased from 20.85% to 70.31% in just 10 min at 30 °C as the concentration of C_4_H_6_O_6_ increased from [C_4_H_6_O_6_] = 2 g/L to [C_4_H_6_O_6_] = 10 g/L; this improved by about 50 percent. With the increase in the concentration of C_4_H_6_O_6_, the number of reactive compounds in the system increased gradually, which promoted the reaction and strengthened the reduction of Cr (VI).

The above results show that the Cr (VI) species could affect the reduction process, and thus, the effect of concentration of H_2_SO_4_ ([H_2_SO_4_] = 0, 0.05, 0.10, 0.15, and 0.20 M) on the reduction process was also investigated. Comparing the results shown in Figure 2 and Figures S1–S4, the concentration of H_2_SO_4_ showed a significant effect. Only 40.63% of the Cr (VI) was reduced at 30 °C for about 80 min at [H_2_SO_4_] = 0 M, while it increased to 100% at the same reaction conditions at [H_2_SO_4_] = 0.20 M. Meanwhile, a reduction efficiency of 100% was obtained in only 25 min at 30 °C with [C_4_H_6_O_6_] = 10 g/L, and in less than 5 min at 90 °C with [C_4_H_6_O_6_] = 10 g/L. A high level of acidic medium could significantly reduce the reaction time and achieve a high reduction efficiency. The effect was similar with the reaction temperature and concentration of [C_4_H_6_O_6_].

Above all, it was concluded that all factors had positive effects on the reduction process, high reaction temperature, high concentration of C_4_H_6_O_6_, and high acidic medium and were all beneficial for the reduction of Cr (VI).

### 2.3. Response Surface Methodology

The response surface methodology analysis (RSM) was applied to optimize the reaction conditions [35,36,37]. In this study, RSM was conducted using Design Expert 8.0 software. The independent variables and factor levels are detailed in Table 2. The reduction efficiency of Cr (VI) was set as the response. The detailed experimental conditions and experimental results are displayed in Table 3.

The simulated results can be seen in Table 3. The square-root-type model was selected to express the model as Equation (4).

Sqrt (η) = 9.80 + 0.59 × A + 0.29 × B + 0.50 × C + 0.54 × D − 0.18 × A × B − 0.34 × A × C − 0.49 × A × D − 0.21 × B × C − 0.39 × B × D − 0.55 × C × D − 0.31 × A × A − 0.092 × B × B − 0.15 × C × C − 0.26 × D × D(4)

**Figure 3:**
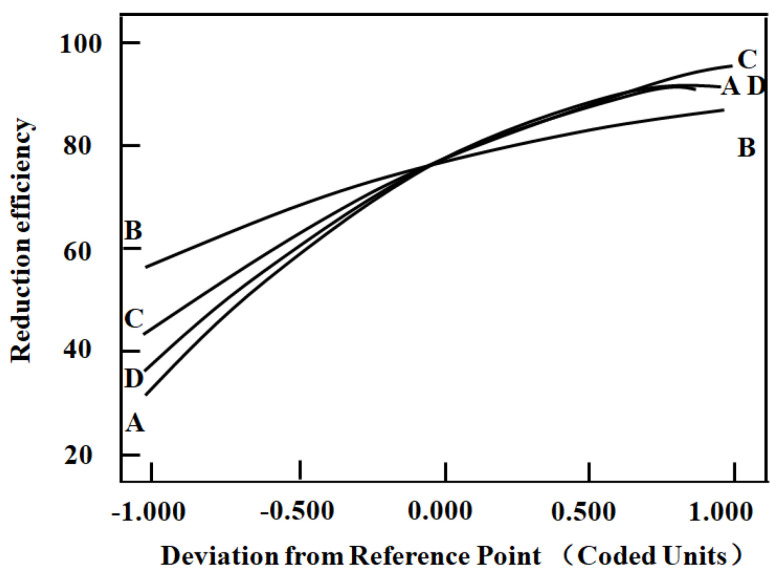
A perturbation plot for the reduction efficiency of Cr (VI) (A: [H_2_SO_4_], B: time, C: temperature and D: [C_4_H_6_O_6_]).

According to the results shown in Equation (5) and Figure 3, the positive coefficients (0.59 (A), 0.29 (B), 0.50 (C), and 0.54 (D)) indicate a significant positive effect on the response. The influence on the reduction process decreased in the following order: [H_2_SO_4_] > [C_4_H_6_O_6_] > reaction temperature > reaction time.

The reduction process of Cr (VI) using C_4_H_6_O_6_ through various variables can be investigated using these model equations. Different parameters, R^2^, *p* values, F values, and adjusted R^2^ values were measured as standards that were helpful for determining the accuracy of every coefficient to evaluate the significance of the predicted model. The ANOVA results (shown in Table 4) confirmed that the Model F value of 37.22 implies that the model was significant. There was only a 0.01% chance that a large “Model F value” could occur due to noise. Values of “Prob > F” less than 0.0500 indicated that the model terms were significant. In this case, A, B, C, D, AC, AD, BC, BD, CD, A^2^, C^2^, and D^2^ were significant model terms. The R^2^ value reflected how much variability in the observed response values could be expressed by the experimental factors as well as their interactions by establishing a relationship between the predicted and experimental results. An R^2^ close to one revealed good fitting of the experimental data to the predicted model equation. The regression model produced a higher R^2^ of up to 0.9738, signifying excellent fit between the model and the experimental data. The predicated R^2^ of up to 0.8493 was in reasonable agreement with the adjusted R^2^ of 0.9477. Adequate precision was helpful for evaluating the signal-to-noise ratio. A ratio greater than 4 was desirable. Here, a higher adequate precision of 19.930 revealed an adequate signal. This regression model could be applied to navigate the design space.

**Figure 4:**
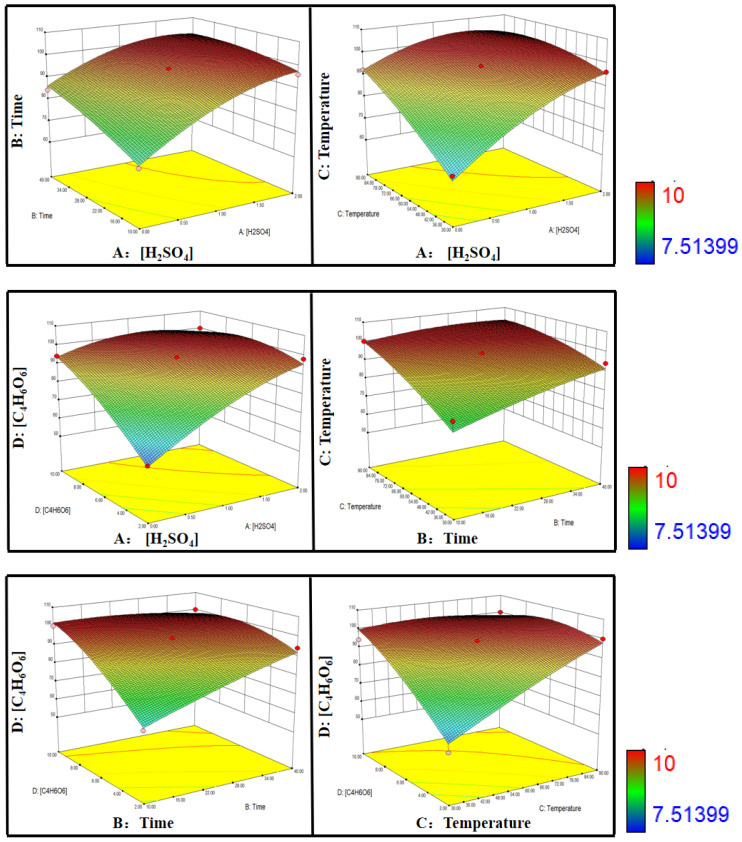
3D counter plots of experimental parameters over reduction efficiency.

Figure 4 describes 3D counter plots of the combined influence of the four experimental parameters over the reduction efficiency. The counter plots provide the mutual interactions between the independent parameters. These response surface plots confirm the perfect and strong interactions between the selected independent experimental parameters. The reduction efficiency was significantly affected by [H_2_SO_4_] and [C_4_H_6_O_6_], while the effect of reaction time was minimal. A high reaction efficiency was easily obtained in the strong acidic medium with a high concentration of C_4_H_6_O_6_ at a high temperature.

### 2.4. Reduction Kinetics Analysis

**Figure 5:**
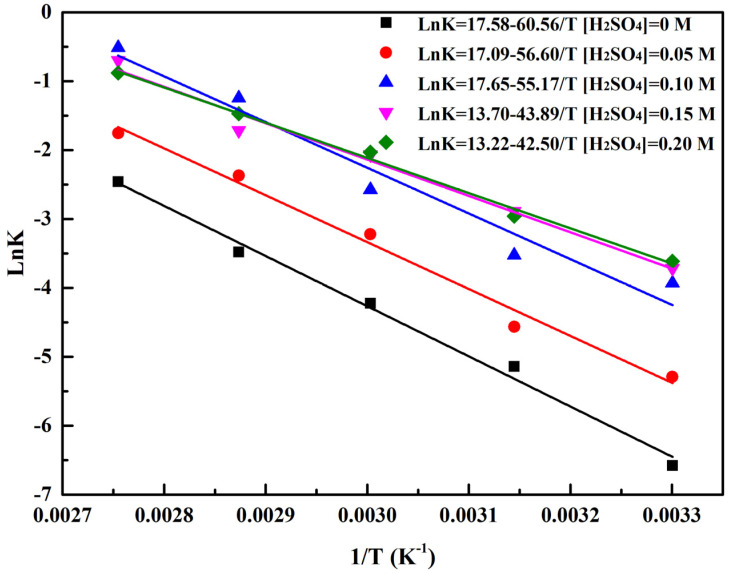
Arrhenius equation for reduction of Cr (VI) at various concentrations of H_2_SO_4_.

To better understand the reaction mechanism, the reduction kinetics behavior was fitted by the pseudo-first-order model [38,39] and the *Ea* for the Cr (VI) reduction based on the Arrhenius equation as in Equation (5) was also calculated. The *Ea* for the Cr (VI) reduction was varied by the reaction conditions. The results shown in Figure 5 indicate that the *Ea* was reduced along with [H_2_SO_4_], which meant that strong acidic medium was beneficial for Cr (VI) reduction. The results are consistent with analysis above.

LnK = LnA-*Ea*/RT (5)

## 3. Experimental Procedure

### 3.1. Materials

The experimental components including K_2_Cr_2_O_7_, H_2_SO_4_ (purchased from Kelong Co., Ltd., Chengdu, China), and tartaric acid (C_4_H_6_O_6_) (purchased from Shanghai Aladdin Biochemical Technology Co., Ltd., Shanghai, China) were used without further purification.

### 3.2. Experimental Procedure

The Cr (VI) solution was prepared by dissolving amount of K_2_Cr_2_O_7_ in distilled water. Firstly, 100 mL of 0.01 M K_2_Cr_2_O_7_ solution was added to a 250 mL beaker placed in a water bath. After the temperature of the water bath had reached the desired temperature, amounts of C_4_H_6_O_6_ and H_2_SO_4_ were added and then stirred at 500 rpm. During the experimental process, the beaker was sealed with cling film to avoid evaporation. A total of 2 mL of solution was collected to measure the residual concentration of Cr (VI) every 5 or 10 min by ICP-OES. Triplicate experiments were performed each time, and measurements accepted if all results were within 10% of the average value. The reduction efficiency (η) was calculated following Equation (6):

(6) $$\eta =\frac{C_{0}-C_{t}}{C_{0}}\times 100\%$$

where C_0_ and C_t_ are the concentrations of Cr (VI) at initial and time t, respectively, in mg/L.

## 4. Conclusions

Small molecular organic polycarboxylate that possessed -COOH and α-OH groups, which had reducing properties and strong complexional ability and were eco-friendly, were widely applied in the reduction process. This paper focused on the reduction process of Cr (VI) and tartaric acid (C_4_H_6_O_6_), which had two -COOH and two α-OH groups. The following conclusions can be reached:

1. Tartaric acid was an efficient reductant for Cr (VI) reduction in a strong acidic medium. Higher concentrations of tartaric acid and higher reaction temperatures could facilitate the reduction process and reduce reaction time.
2. A total of 100% of the Cr (VI) could be reduced by C_4_H_6_O_6_ in a strong acidic environment under high reaction temperatures. Response surface methodology analysis confirmed that the experimental parameters had a positive effect on the reduction process and followed the order [H_2_SO_4_] > [C_4_H_6_O_6_] > reaction temperature > reaction time.

## Figures and Tables

**Table 1:** Treatment technologies for chromium removal [16].

No.		Methods	Advantages	Disadvantages
1	Physicochemical Processes	Chemical Precipitation	simple, effective	secondary pollution
2		Membrane	higher removal efficiency, no pollution loads and sometimes lower energy consumption	highly depend on materials, membrane pore size, and composition
3		Ion Exchange	high efficiency, low cost, less sludge volume, and high selectivity	highly depends on resin structure and the solution environment
4		Adsorption	high efficiency, simple operation and ease of regeneration	highly depends on the solution environment
5	Electrochemical Technologies	Electrocoagulation	simple, productive, ease of operation	poor systematic reactor design and sacrifice of electrodes
6		Electrochemical Reduction	no further reagent	dependent on the electrode materials and electrochemical surface area of the electrode
7		Electrodialysis	low energy consumption	high cost of electrodes
8	Advanced Technologies	Photocatalysis	simple design, low-cost operation, high stability, and high removal efficiency	producing unwanted byproducts
9		Nanotechnology	higher removal efficiency, low waste generation, and specific uptake	increase the risk of nano-pollutants in the environment

**Table 2:** Independent variables and factor levels.

Independent Variable	Unit	Level		
		−1	0	1
A: [H_2_SO_4_]	mol/L	0	0.1	0.2
B: Time	min	10	25	40
C: Temperature	°C	30	60	90
D: [C_4_H_6_O_6_]	g/L	2	6	10

**Table 3:** The reaction conditions.

Run	A: [H_2_SO_4_]	B: Time	C: Temperature	D: [C_4_H_6_O_6_]	Actual Reduction Efficiency (%)
1	1	25	60	6	96.04
2	0	40	60	6	84.17
3	1	40	60	2	94.06
4	1	25	90	2	100.00
5	1	40	90	6	100.00
6	1	10	60	10	100.00
7	1	25	60	6	96.04
8	1	25	90	10	100.00
9	2	25	30	6	96.04
10	1	40	30	6	94.06
11	1	25	60	6	96.04
12	0	25	30	6	64.38
13	1	10	30	6	78.23
14	0	10	60	6	68.34
15	0	25	60	10	94.06
16	2	10	60	6	96.04
17	2	40	60	6	100.00
18	1	25	30	10	94.06
19	2	25	60	10	10.00
20	1	25	60	6	96.04
21	1	10	90	6	100.00
22	2	25	90	6	100.00
23	0	25	90	6	92.08
24	0	25	60	2	58.44
25	2	25	60	2	98.02
26	1	25	30	2	56.46
27	1	40	60	10	100.00
28	1	10	60	2	66.36
29	1	25	60	6	96.04

**Table 4:** Analysis of variance (ANOVA) for the response.

Source	Sum of Squares	Df	Mean Square	F Value	*p* Value Prob > F
Model	16.19	14	1.16	37.22	<0.0001
A	4.20	1	4.20	135.06	<0.0001
B	1.03	1	1.03	33.11	<0.0001
C	3.02	1	3.02	97.07	<0.0001
D	3.51	1	3.51	113.06	<0.0001
A × B	0.13	1	0.13	4.03	0.0645
A × C	0.47	1	0.47	15.15	0.0016
A × D	0.95	1	0.95	30.73	<0.0001
B × C	0.18	1	0.18	5.86	0.0296
B × D	0.60	1	0.60	19.39	0.0006
A × A	0.62	1	0.62	19.80	0.0005
B × B	0.055	1	0.055	1.78	0.2032
C × C	0.14	1	0.14	4.64	0.0491
D × D	0.43	1	0.43	13.94	0.0022
Residual	0.021	7	0.00294	-	-
Lack-of-fit	0.44	10	0.044	-	-
Pure error	0.000	4	0.000	-	-
Cor Total	16.63	28			
R-Squared	0.9738				
Adj-Squared	0.9477				
Pred R-Squared	0.8493				

## Data Availability

Data are contained within the article and Supplementary Materials.

## Supplementary Materials

The following supporting information can be downloaded at: https://www.mdpi.com/article/10.3390/molecules29225459/s1. Figure S1: The effect of experimental parameters on the reduction efficiency of Cr (VI) at [H_2_SO_4_] = 0.05 M; Figure S2: The effect of experimental parameters on the reduction efficiency of Cr (VI) at [H_2_SO_4_] = 0.10 M; Figure S3: The effect of experimental parameters on the reduction efficiency of Cr (VI) at [H_2_SO_4_] = 0.15 M; Figure S4: The effect of experimental parameters on the reduction efficiency of Cr (VI) at [H_2_SO_4_] = 0.20 M.
